# Taxonomic Profiling of Bacterial Communities Associated with Metals in the Tar Mats and Cyanobacterial Mats of the Qatar Coast, Arabian Gulf

**DOI:** 10.1007/s00248-026-02761-y

**Published:** 2026-04-24

**Authors:** Kaiprath Puthiyapurayil Haseeba, Kesava Priyan Ramasamy, Ghada Mustafa, Subramanian Veerasingam, Valliyil Mohammed Aboobacker, Cheriyeri Poyil Abdulla, Jassim Abdulla Al-Khayat, Ponnumony Vethamony

**Affiliations:** 1 UNESCO Chair in Marine Sciences, Environmental Science Center, Qatar University, P.O. Box: 2713, Doha, Qatar; 2 Department of Ecology and Environmental Science, Umeå University, Umeå, 90187 Sweden; 3 Department of Biological and Environmental Sciences, Qatar University, P.O. Box: 2713, Doha, Qatar

**Keywords:** Tar mat, Bacteria, Metals, 16S amplicon sequencing, Taxonomy, Arabian Gulf

## Abstract

Tar mats are the final weathered petroleum residues derived from natural or human-induced incidents that form mats of varying sizes along the intertidal or coastal zones following prolonged environmental exposure. This study investigates the bacterial communities associated with tar mats from two distinct coastal areas of Qatar: Ras Rakan (an uninhabited island) and Al Khor (an anthropogenically influenced coast), using 16 S rRNA gene sequencing of the V3–V4 regions. The results showed the dominance of Proteobacteria across all samples, and notably higher relative abundances of *Thermovirga*, *Salinivibrio*, *Synechococcus* IR11, and *Arcobacter* in samples from Ras Rakan compared to Al Khor. Elemental analysis of tar mat samples from Ras Rakan showed notable differences in concentrations across sample types, with higher concentrations of the major elements Ca, Mg, Al, and Fe, as well as petroleum-related elements such as Cu, Ni, and V. The occurrence of genera is probably linked to high concentrations of heavy metals, enabling bacteria to respond to the tar-contaminated environment. Positive correlations were observed between heavy metals and several taxa, including *Synechococcus* spp., highlighting the potential role of cyanobacteria in metal biosorption and hydrocarbon-associated cyanobacterial consortia. The correlation between these metals and cyanobacterial diversity highlights the importance of metal-microbe interactions in understanding the ecological consequences of oil contamination in coastal environments.

**Graphical Abstract:**

**Figure:**
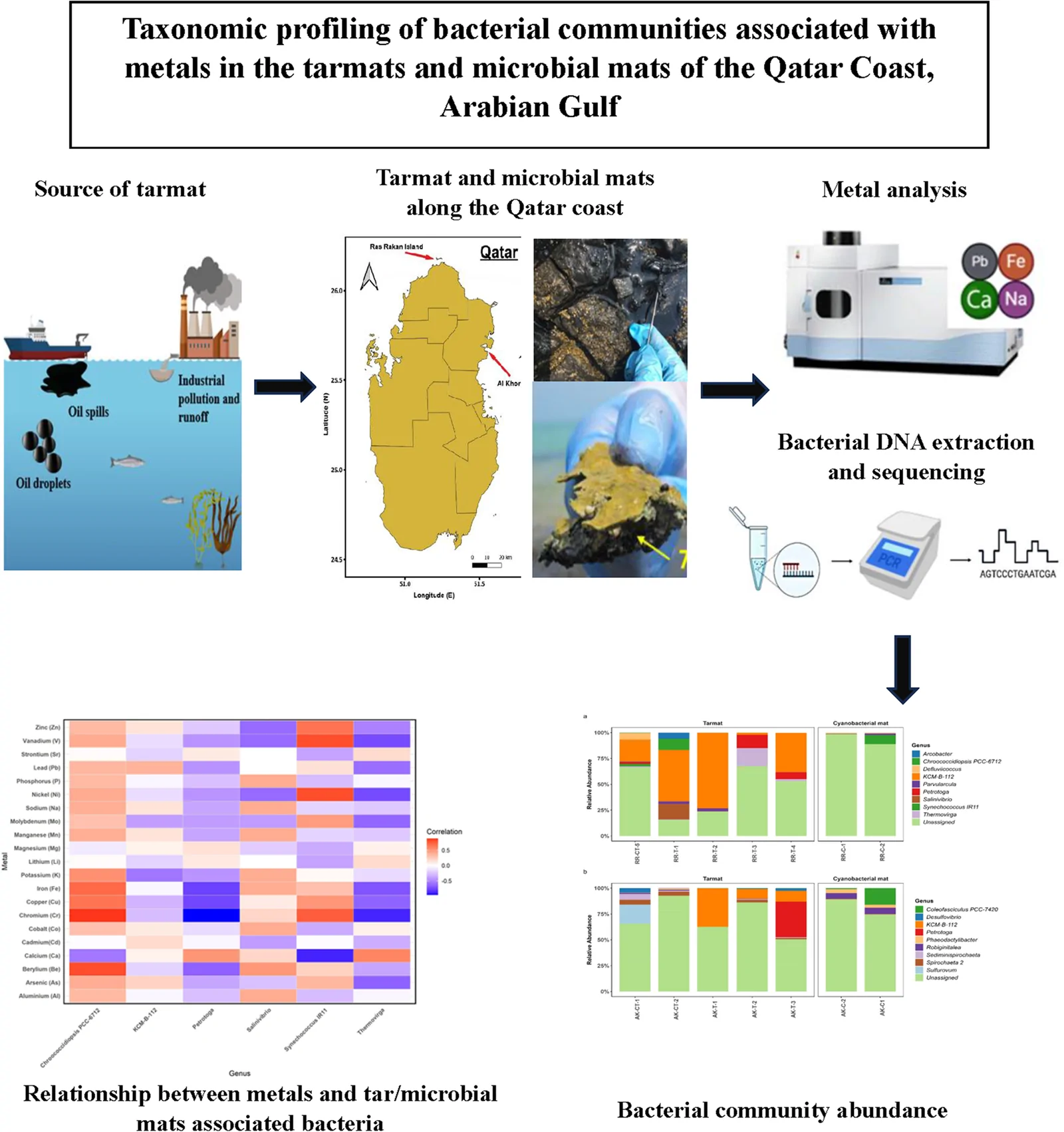

**Supplementary Information:**

The online version contains supplementary material available at 10.1007/s00248-026-02761-y.

## Introduction

The Arabian Gulf (“the Gulf”) holds global significance in hydrocarbon production, which is foundational to its regional economy, as the region is heavily involved in oil extraction and related activities. The Gulf has been severely impacted by two major catastrophic events, the Nowruz Field oil spill in 1983 and the Gulf War oil spill in 1991 that eventually led in the occurrence of tar mats along the coast. Tar is a type of oil residue that originates from natural and anthropogenic marine oil spills and is deposited as tar mats or tar balls after undergoing several weathering processes. The time required for tar formation may vary from a few days to several months, depending on wave intensity, currents, and winds during oil transport through seawater [1]. Nearly 10.8 million barrels of crude oil were released during the 1991 Gulf War, and subsequent studies documented oil spill contaminants at various beaches along the Qatar coast. Recently, Arekhi et al. [2] identified tar mats deposited on the northern beaches of Qatar that closely matched the fingerprints of Kuwait crude oil, concluding that the tar residues originated from the 1991 Gulf War oil spill.

. Oil pollution is considered as a potential source of heavy metal contamination in the Gulf waters. Primary heavy metals notably linked to oil spill incidents in the region include lead (Pb), nickel (Ni), vanadium (V), zinc (Zn), and cadmium (Cd), mainly originating from dredging, sewage discharge, and industrial effluents, particularly from desalination plants [3]. Metals may hinder natural biodegradation by directly interacting with cyanobacterial enzymes during the process or by disrupting enzymes involved in general cellular metabolism [4]. Most metals exhibit toxicity to microbes at specific concentrations, and inhibition of bacterial growth due to high concentrations of heavy metals has been documented [5] Phylogenetically diverse cyanobacterial communities associated with tar residues have been documented. Recent studies have isolated tar-associated bacteria, identifying potential hydrocarbon degraders such as *Halomonas*, *Pseudomonas*, and *Marinobacter*, along with potential human pathogens including *Acinetobacter*, *Klebsiella*, *Rhodococcus*, *Staphylococcus*, and *Vibrio* [6, 7]. Well-developed cyanobcaterial mats have also been observed along the Qatar coastline in the intertidal zone close to mangrove areas, characterized by the cyanobacterial species *Calothrix*, *Anabaena*, *Phormidium*, *Microcoleus*, *Oscillatoria*, *Lyngbya*, and *Scytonema* [8]. The growth of cyanobacterial mats on oil-polluted sediments, exhibiting high cyanobacterial diversity and diverse metabolic pathways, has been proposed as an initial step toward natural biodegradation [9]. However, a knowledge gap exists regarding the presence of cyanobacterial communities and their potential functions associated with tar mats in the Gulf region. The current study focuses on two sites impacted by tar mats: Ras Rakan and Al Khor. Ras Rakan is a small, ‘T’-shaped, uninhabited island approximately 2 km north of Qatar’s mainland, characterized by a low-lying, grass-covered landscape. Al Khor is a coastal city located about 50 km north of Doha, and is known for its natural harbor, Al Khor Bay, which extends into the Gulf. The city has seen significant industrial expansion in recent years, particularly in the petroleum and natural gas sectors, and is also home to a substantial natural mangrove forest.

To the best of our knowledge, no studies have yet correlated elemental concentrations in tar residues with associated cyanobacterial community structure and abundance. In addition, comparing the cyanobacterial communities of tar mats and naturally occurring cyanobacterial mats could advance bioremediation strategies. For the present study, both tar mats and cyanobacterial mats were collected from identical locations within each site to minimize spatial heterogeneity and facilitate the assessment of potential correlations between cyanobacterial community structure and elemental concentrations. Therefore, a wide range of tar mat types - soft, hard, hydrocarbon-layered, and plastic-embedded were sampled to understand the differences in structural characteristics of tar mats. It is anticipated that differences in elemental composition among these tar mat types could influence cyanobacterial diversity by imposing selective pressures that either inhibit or enrich specific taxa, particularly hydrocarbon-degrading bacteria. Hence, the present study was taken up with the following objectives: (i) to investigate the bacterial communities associated with both tar mats and cyanobacterial mats of the northern part of Qatar, based on amplicon sequencing of the V3–V4 region of the 16 S rRNA gene, and (ii) to elucidate the influence of elemental concentrations on the bacterial community structure in the tar mat and cyanobacterial mat samples.

## Materials and Methods

### Study Area and Sample Collection

The Gulf is 1000 km long and 338 km wide, with an average depth of approximately 36 m [10]. The Qatar peninsula covers approximately 11,500 sq km and is situated in the southwestern region of the Gulf. Qatar has a subtropical arid climate defined by moderate winters and extremely hot summers. For the present study, Ras Rakan and Al Khor were selected as tar mat-impacted sites due to their distinct characteristics. Ras Rakan is an uninhabited island, while Al Khor is a coastal city that has witnessed substantial industrial development in recent years and is located near the prominent oil and gas fields of the country.

**Fig. 1:**
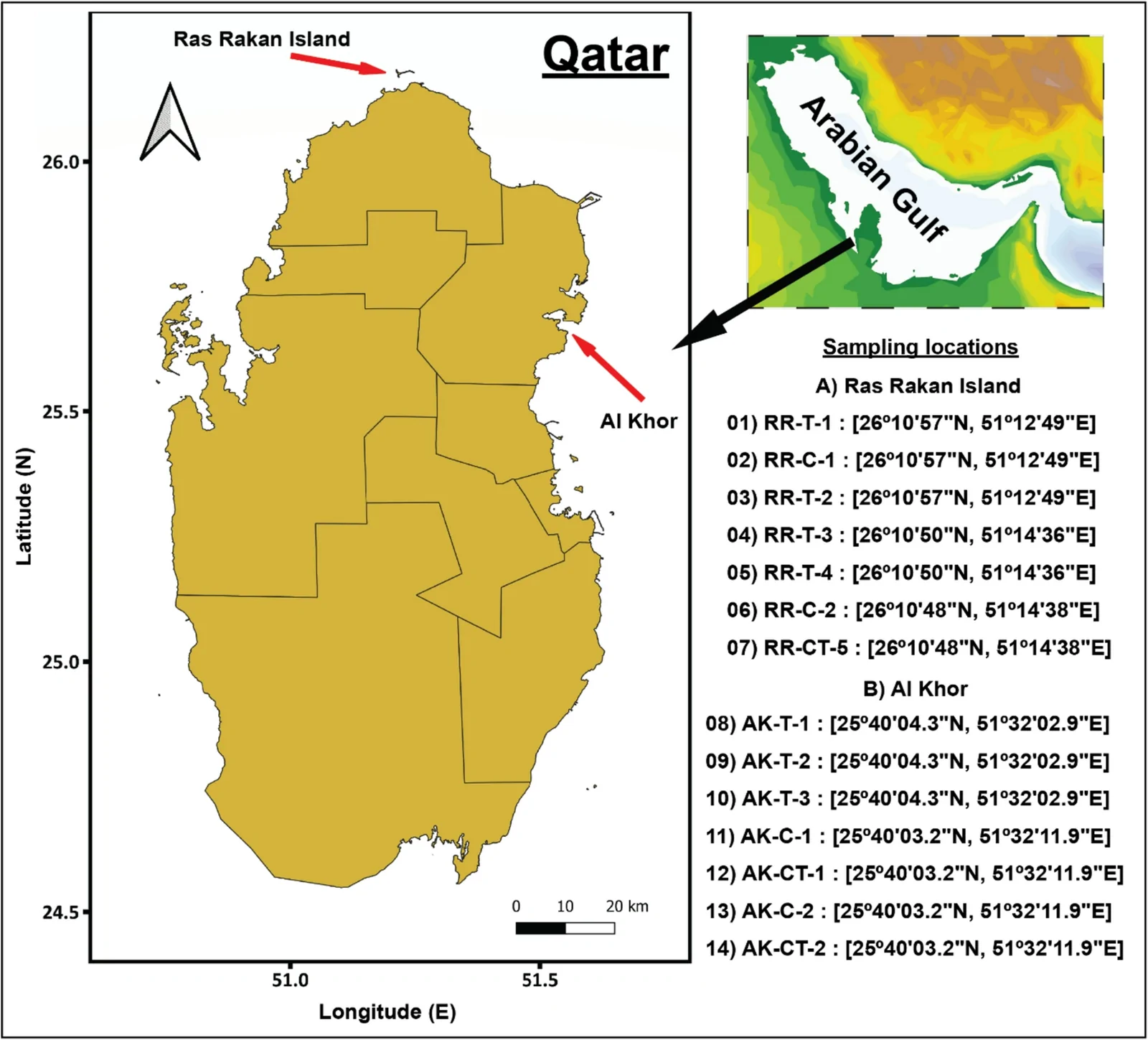
Study area map of Qatar coast displaying along with sampling coordinates of Ras Rakan Island and Al Khor

Tar mat andcyanobacterial mat samples were collected from Al Khor and Ras Rakan Island during late summer of 2018 and 2020, respectively (Fig. 1; Table 1). The samples were collected with a sterilized stainless-steel spatula, stored in labeled bags, and subsequently stored at −20 °C. A total of 14 tar mat samples were collected, seven from each site (Fig. S1, Fig. S2). In addition, a portable pH/DO/temperature meter (YSI EXO 2 multiparameter sonde) is used at each site to measure the physico-chemical characteristics of water from the Al Khor and Ras Rakan regions.

**Table 1:** Sampling locations, coordinates and sample description

Sample Location	Coordinates	Sample code	Sample type	Sample Description
Ras Rakan Island	26º10’57"N 51º12’49"E	RR-T-1	Tar mat	Soft tar mat attached to rocks
		RR-C-1	Cyanobacterial mat	Cyanobacterial mat from seawater
		RR-T-2	Tar mat	Hard tar mat attached to rocks
Ras Rakan Island	26º10’50"N 51º14’36"E	RR-T-3	Tar mat	Soft tar mat attached to rocks
		RR-T-4	Tar mat	Tar mat embedded with plastic
Ras Rakan Island	26º10’48"N 51º14’38"E	RR-C-2	Cyanobacterial mat	Cyanobacterial mat imposed on hydrocarbon layer from seawater
		RR-CT-5	Tar mat	Hydrocarbon layer below the cyanobacterial layer (RR-C-2) from seawater
Al Khor	25º40’04.3"N 51º32’02.9"E	AK-T-1	Tar mat	Tar mat attached to beach rocks
		AK-T-2	Tar mat	Tar mat attached to corals
		AK-T-3	Tar mat	Tar mat covered by beach sands
Al Khor	25º40’03.2"N 51º32’11.9"E	AK-C-1	Cyanobacterial mat	Cyanobacterial mat near mangrove roots
		AK-CT-1	Tar mat	Hydrocarbon layer below the cyanobacterial layer (AK-C-1) near mangrove root
		AK-C-2	Cyanobacterial mat	Cyanobacterial mat from seawater
		AK-CT-2	Tar mat	Hydrocarbon layer below the cyanobacterial layer (AK-C-2) from seawater

### Elemental Analyses by ICP-OES

Elemental analysis in tar mat and cyanobacterial mat samples collected from Ras Rakan followed the methods described by [11]. Briefly, 5 g of tar residues were treated with 3 ml of concentrated H_2_SO_4_, heated to 130 °C for 1 h, and subsequently ashed for roughly 6 h in a muffle furnace at 550 °C. The cooled ash (0.3 g from each sample) was transferred into a Polytetrafluoroethylene (PTFE) Teflon digestion tube and treated with concentrated HNO_3_, sequentially heated at 95 °C, 135 °C, and 150 °C until near dryness. The digest was treated with 0.3 ml of H_2_O_2_ and 1 ml of HNO_3_, diluted with 25 ml Milli-Q water, filtered, and analyzed using ICP-OES (Inductively Coupled Plasma Optical Emission spectroscopy (Perkin Elmer Optima 7300DV System). The concentration of 21 elements were: Al, As, Be, Ca, Cd, Co, Cr, Cu, Fe, K, Li, Mg, Mn, Mo, Na, Ni, P, Pb, Sr, V, and Zn. Accuracy of the method was assessed using the certified reference material CRM-PACS-3 and recovery values ranging from 89 to 101%.

Elemental and bacterial analyses were performed for the samples collected from Ras Rakan. However, due to logistical issues, bacterial analyses could only be carried out for the samples from Al Khor.

### DNA Extraction, Amplicon Sequencing, and Bioinformatics

The seven tar mat samples collected from the Ras Rakan Island and Al Khor were used for DNA extraction. For DNA extraction, samples were thawed for 30 min before processing. Around 0.5 g from each sample was used for DNA extraction using QIAGEN DNeasy PowerSoil Kit. DNA was amplified by PCR using the primers 16 S variable (V3-V4) region [forward (341 F) and reverse (805R) primers (v341F - CCTACGGGNGGCWGCAG and 805R - GACTACHVGGGTATCTAATCC)]. All PCR reactions were performed in triplicate. Nuclease-free water was used as a negative control. The initial denaturation temperature was set to 95 °C for 3 min, followed by 25 cycles: 95 °C for 30 s, 55 °C for 30 s, and 72 °C for 30s. The final extension temperature was set to 72℃ for 7 min. Library preparation and sequencing were performed according to standard protocols at Macrogen Inc., South Korea. Paired-end sequences were trimmed, assembled, and denoised using the dada2 plugin of the QIIME2 platform [12]. Filtration, chimera removal and clustering were also done using the same platform. For bacterial taxonomic assignment, the SILVA v. 138.1 database was used; the classifier was trained using the feature-classifier plugin [13]. Classification was done at 99% similarity, and assignment was done using QIIME2 classify-sklearn default settings. Operational Taxonomic Units (OTUs) classified at each level were used to generate correlation matrices that visualize relationships among taxa. Principal coordinate analysis (PCA) was performed on Euclidean distance matrices of bacterial abundance data, and plots were generated using ggplot2 in R [14]. Alpha diversity (Shannon and Species richness) was calculated using the vegan community ecology package [15]. Permutational analysis of variance (PERMANOVA) using the adonis function in the vegan R package was performed to determine significant differences in bacterial community composition. The Wilcoxon rank-sum test was used to compare the relative abundances and alpha diversity metrics across sample types. Correlation coefficient analysis was performed in R using the Spearman correlation to assess relationships between metal concentrations and the relative abundance of the top 10 genera in Ras Rakan.

The raw nucleotide sequence dataset was deposited in the NCBI Sequence Read Archive (SRA) under the accession number PRJNA714822.

## Results

### Physico-chemical Parameters of Seawater in Al Khor and Ras Rakan

The seawater physico-chemical parameters were measured at Al Khor and Ras Rakan, as summarized in Table S1. The sea surface temperature remained relatively stable in both, ranging from 32.62 to 33.68 °C. Dissolved oxygen (DO) varied between 6.21 mg/L and 6.70 mg/L, with the highest value at the Al Khor site 1. The pH exhibited minimal fluctuations, remained alkaline (7.95–8.54), and salinity ranged from Ras Rakan (43.57) to Al Khor (42.85). In general, sea surface temperatures along the coast of Qatar may range from 18 to 22 °C in winter to 31 to 36 °C in summer [16]. The seawater salinity ranges from 39 to 46 and can even exceed 60 in regions like the Gulf of Salwa [17]. Chlorophyll-a levels were lower in both sites, with Al Khor exhibiting higher values ranging from 0.59 to 1.04 (µg/L) compared to Ras Rakan. Overall, the in situ physico-chemical parameters were relatively stable across the two regions, except for a higher turbidity range in the Al Khor region (14.76–50.61 NTU), likely due to sediment resuspension during sampling.

**Fig. 2:**
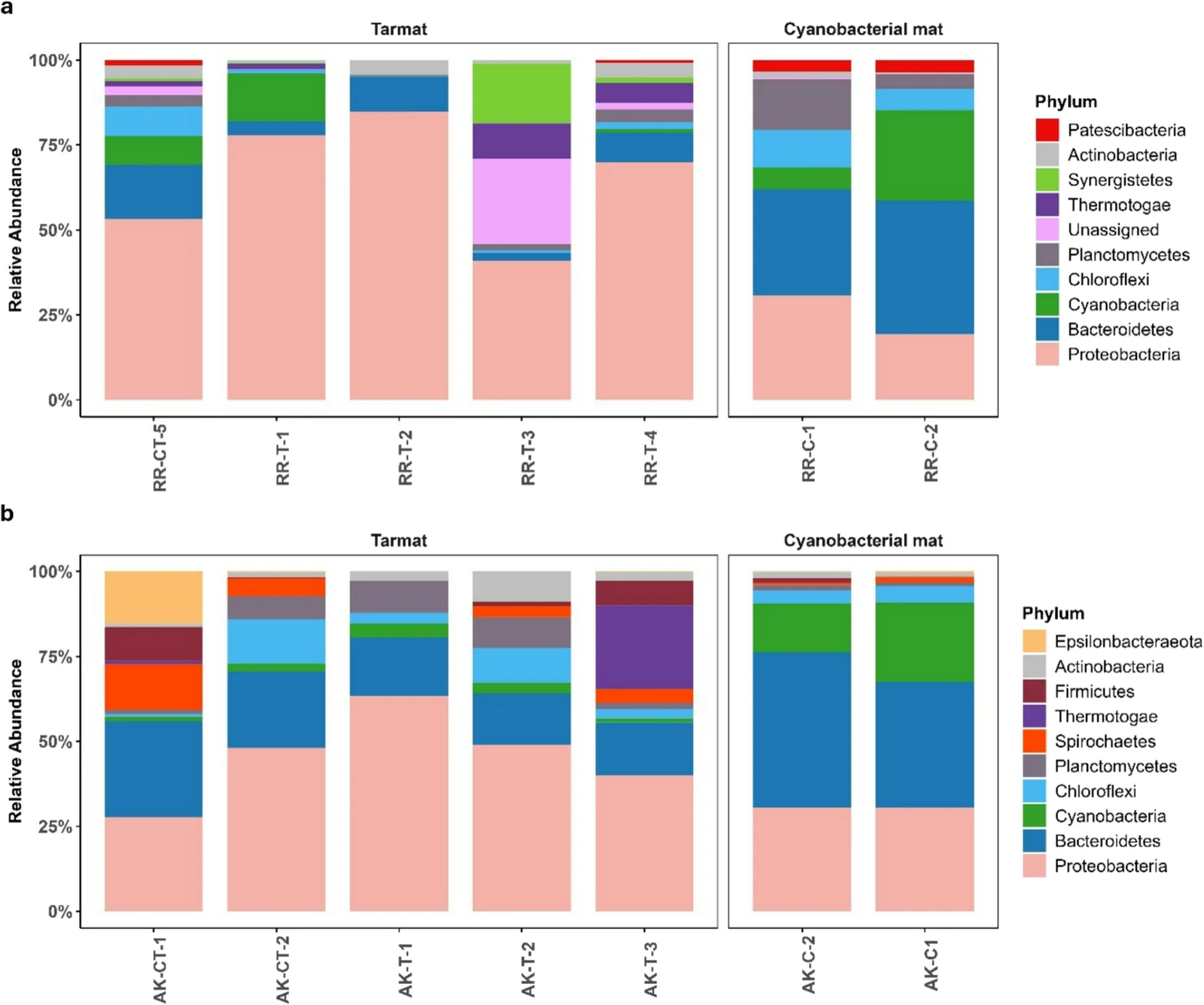
Bar chart showing relative abundance percentage of top10 bacterial phyla from the samples collected (**a**) Ras Rakan Island (**b**) Al Khor. Panels represent: (**a**) Ras Rakan Island and (**b**) Al Khor. Within each panel, individual bars corresponds to each samples collected from tar mat and cyanobacterial mat

At Ras Rakan, samples comprised both hard (RR-T-2) and soft (RR-T-1, RR-T-3) tar mats, alongside plastic debris embedded within the tar mats (RR-T-4). Two cyanobacterial mats (RR-C-1 and RR-C-2) displayed a distinct hydrocarbon-rich layer (RR-CT-5) (Fig. 2). From Al Khor, two types of tar mat residues were collected: hard tar mats adhering to beach rocks (AK-T-1) and corals (AK-T-2, AK-T-3), as well as semi-hard, sand-embedded tar mats (AK-T-3). cyanobacterial mats sampled around mangrove roots contained a cyanobacterial layer (AK-C-1) overlain by black and grey hydrocarbon layers (AK-CT-1). Additionally, two cyanobacterial mats from shallow water exhibited a similar two-layer composition (AK-C-2 and AK-CT-2).

The elemental concentrations were determined for Ras Rakan samples and are presented in Table 2. Notable variations in elemental concentrations were observed among the tar mat and cyanobacterial mat samples, with dominant elements included Ca, Mg, Al, Fe, and K. cyanobacterial mat sample RR-C-1 showed increased concentrations of Al (9738.82 µg g⁻¹), Fe (6839.07 µg g⁻¹), Na (25682.63 µg g⁻¹), K (5274.1 µg g⁻¹), Mg (17934.83 µg g⁻¹), Sr (3188.26 µg g⁻¹), and P (1874.55 µg g⁻¹), alongside toxic and trace elements such as Ni (196.99 µg g⁻¹), As (181.02 µg g⁻¹), Be (178.1 µg g⁻¹), Mo (215.48 µg g⁻¹), Pb (183.38 µg g⁻¹), Co (180.15 µg g⁻¹), and Cr (203.67 µg g⁻¹). In contrast, cyanobacterial mat RR-C-2 exhibited lower concentrations of Cr (6.25 µg g⁻¹), Zn (32.86 µg g⁻¹), V (169.95 µg g⁻¹), and Ni (81.64 µg g⁻¹), while Ca (56,011.29 µg g⁻¹) and Mg (4706.29 µg g⁻¹) had comparatively increased concentration. Among the tar mat samples, soft tar mat RR-T-3 contained the highest Ca concentration (227,205.89 µg g⁻¹), coupled with relatively low levels of Cr (0.22 µg g⁻¹), Mo (2.13 µg g⁻¹), and Ni (11 µg g⁻¹). The hard tar mat RR-T-2 showed moderate concentrations of Ca (127,580.8 µg g⁻¹), Mg (10,581.2 µg g⁻¹), Na (7230.54 µg g⁻¹), Ni (45.71 µg g⁻¹), Cr (2.56 µg g⁻¹), and Mo (6.34 µg g⁻¹). The hydrocarbon-rich layer (RR-CT-5) exhibited the lowest overall elemental concentrations, including Al (7.23 µg g⁻¹), Fe (29.66 µg g⁻¹), Mg (451.95 µg g⁻¹), Na (955.34 µg g⁻¹), K (60.56 µg g⁻¹), and P (15.35 µg g⁻¹), with elements such as As, Be, Cu, and Pb below detection limits.

**Table 2:** Concentration of metals (µg g^− 1^) in the tar mat samples of Ras Rakan Island

Analytes	RR-T-1	RR-C-1	RR-T-2	RR-T-3	RR-T-4	RR-C-2	RR-CT-5
Al	2064.9	9738.82	812.91	1318.28	1619.02	686.33	7.23
Ca	141461.6	82871.02	127580.8	227205.89	222488.81	56011.29	10377.24
Fe	1267.58	6839.07	538.92	247.61	344.01	488.41	29.66
K	1186.83	5274.1	614.95	923.05	635.61	746.38	60.56
Mg	8600.44	17934.83	10581.2	14568.63	15937.91	4706.29	451.95
Na	10709.75	25682.63	7230.54	7209.71	7351.67	7074.06	955.34
P	817.1	1874.55	455.37	654.18	740.09	166.46	15.35
As	nd	181.02	0.5	nd	nd	nd	nd
Be	0.01	178.1	nd	nd	nd	nd	nd
Cd	0.12	181.71	0.35	0.22	0.25	0.13	0.05
Co	0.74	180.15	0.45	0.53	0.59	0.35	0.41
Cr	4.41	203.67	2.56	0.22	0.73	6.25	0.73
Cu	1.32	181.6	1.28	nd	1.32	2.56	nd
Li	1.82	177.77	1.31	2.04	1.94	0.63	0.06
Mn	21.81	273.69	11.11	10.3	12.67	9.77	0.46
Mo	0.88	215.48	6.34	2.13	1.83	11	0.21
Ni	5.31	196.99	45.71	11	13.89	81.64	27.82
Pb	0.48	183.38	3.3	nd	0.83	0.3	nd
Sr	2056.45	3188.26	1396.81	2794.46	2552.83	585.93	109.91
V	5.08	200.93	134.46	35.1	36.92	169.95	82.52
Zn	10.23	187.7	45.12	22.03	28.33	32.86	48.12

nd – not detected

### Taxonomic Composition of Bacterial Communities and Alpha Diversity Measurements

**Fig. 3:**
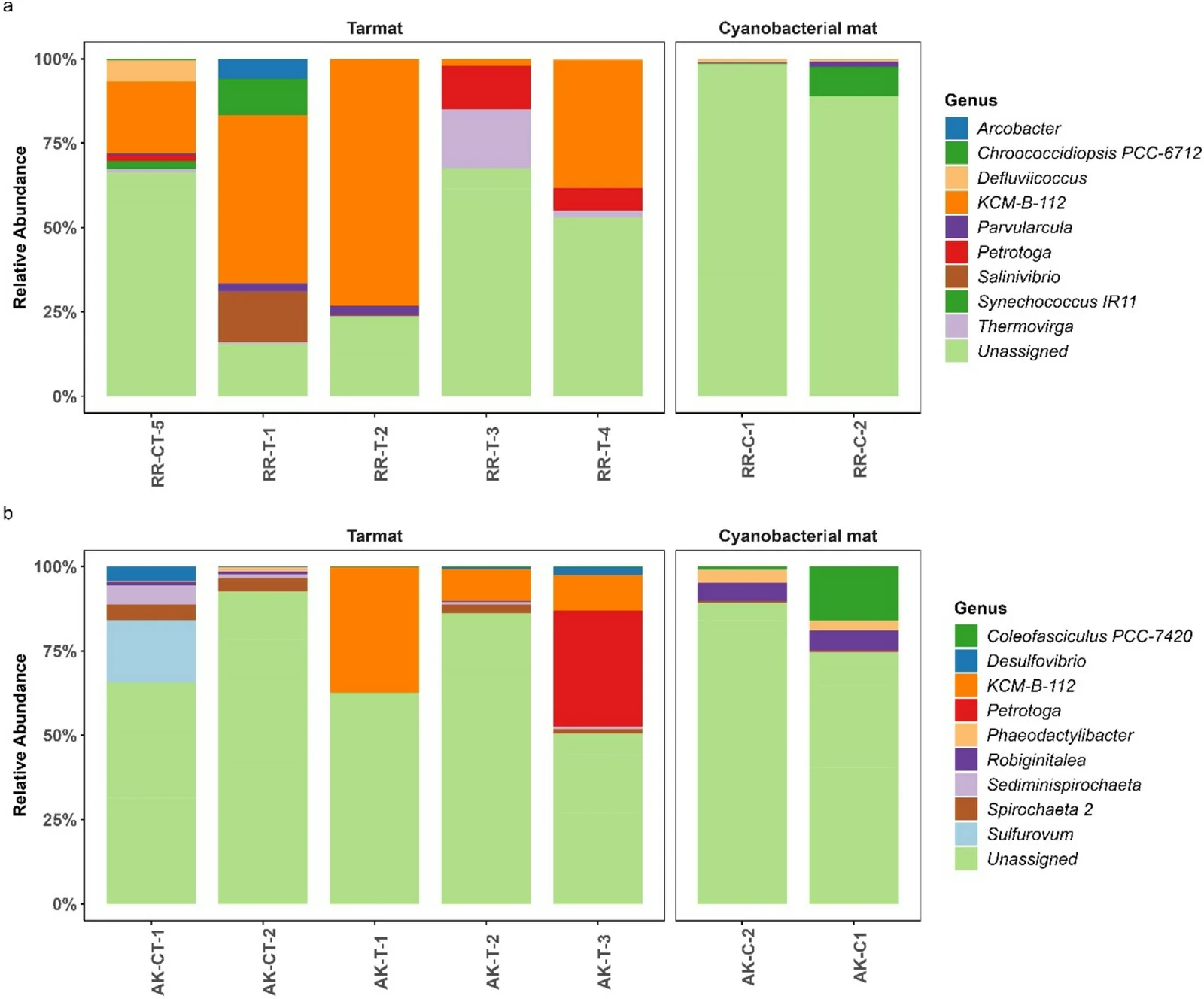
Bar chart showing relative abundance of top10 genus at (**a**) Ras Rakan Island (**b**) Al Khor tar mat and cyanobacterial mat samples

The Proteobacteria phylum was found to be dominant in the tar mat samples from Ras Rakan and Al Khor. The majority of bacterial phyla found in Ras Rakan tar mat samples were Proteobacteria, Bacteriodetes, Cyanobacteria, Synergistetes, Thermotogae, Actinobacteria, Chloroflexi, Patescibacteria and Planctomycetes. In contrast, cyanobacterial mats were dominated by Bacteriodetes rather than Proteobacteria, followed by Planctomycetes, Chloroflexi, Patescibacteria and Actinobacteria. The relative abundance each phyla showed variation in tar mat and cyanobacterial mats however no significant difference at the phylum level. The relative abundance of each phylum varied between the tar mats and the cyanobacterial mats, however, no significant differences were detected at the phylum level (Wilcox rank sum test, *p* > 0.05) (Fig. 2a). Similar to Ras Rakan tar mat samples, Proteobacteria was the dominant phylum in tar mat samples of Al Khor, other phyla present were Bacteroidetes, Chloroflexi, Cyanobacteria, Planctomycetes, Spirochaetes, Thermotogae, Firmicutes, Actinobacteria and Epsilonbacteraeota (Fig. 2b). The cyanobacterial mats in Al Khor samples were dominated by Bacteriodetes and Proteobacteria. The overall relative abundance of bacterial community composition at the phyla level differed significantly between tar mat and cyanobacterial mats (PERMANOVA R² = 0.358, *p* = 0.001) and no significant difference of community composition was observed between Ras Rakan and Al Khor (R² = 0.111, *p* = 0.070). In Ras Rakan, about 49.7% of OTUs are assigned to the KCM-B-112 *Salinivibrio* (15%), Chroococcidiopsis PCC-6712 (10.8%) and Arcobacter (5.9%) in RR-T-1. Similarly, KCM-B-112 had a higher abundance in tar mat sample RR-T-2, while in the cyanobacterial mat samples this genus was found in considerable low abundance (Fig. 3a). A considerable increase in the relative abundance of the genus *Thermovirga* (17.4%) and *Petrotoga* (12.8%) in one of the tar mat (RR-T-3) sample. In the Cyanobacterial mat sample RR-C-2, the genus *Synechococcus* IR11 (8.7%) showed a higher relative abundance than in other Cyanobacterial mat samples. We found no significant difference at the top 10 genus level in both sample types in Ras Rakan (Wilcoxon rank-sum test, *p* > 0.05). The relative abundance of KCM-B-112 was found to be significantly higher in the three tar mat samples, namely, AK-T-1, AK-T-2, and AK-T-3 with percentages of 37.2%, 9.4%, and 10.4%, respectively. The genus *Sulfurovum* was found in a higher abundance (18.3%) in tar mat sample AK-CT-1 from Al Khor. Bacterial genera such as *Sediminispirochaeta*, *Spirochaeta*, *Petrotoga* were found to be dominant in tar mat samples from Al Khor (Fig. 3b). In the Cyanobacterial mat sample AK-C-2 from Al Khor, genera such as *Robiginitalea*,* Phaeodactylibacter*,* Coleofasciculus* PCC-7420, were higher relative abundance of 5.5%, 3.8% and 0.8%. However, in AK-C-1, the relative abundance of *Coleofasciculus* PCC-7420 was 15.9% (Fig. 3b). The relative abundances of the top 10 genera between the two sample categories (tar mat and cyanobacterial mat) were found no significant differences (Wilcoxon rank-sum test, *p* > 0.05).

**Fig. 4:**
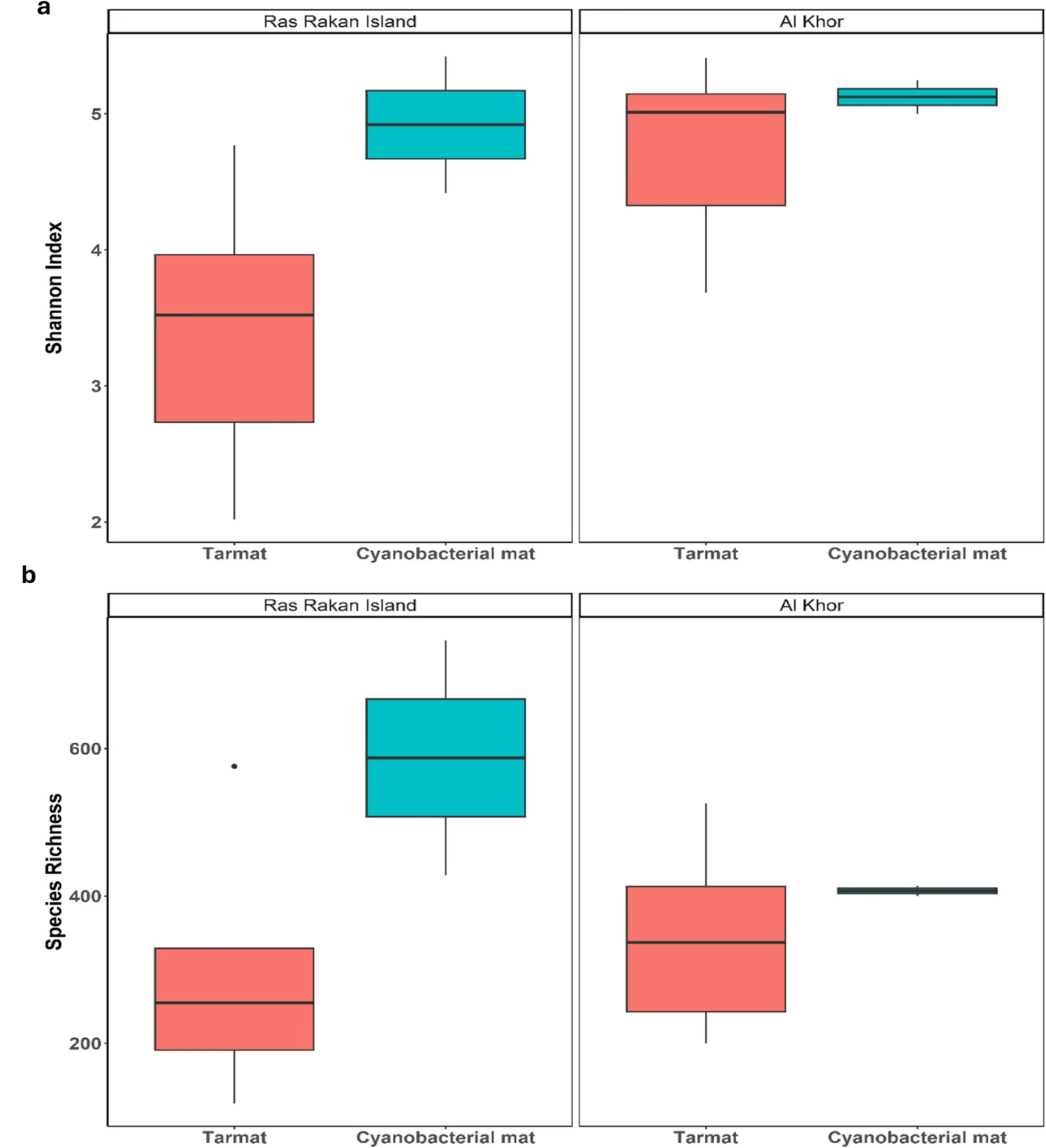
Alpha diversity measurements (**a**) Shannon index (**b**) Species richness of 16 S bacterial communities from Ras Rakan Island and Al Khor tar mat and cyanobacterial mat samples

**Fig. 5:**
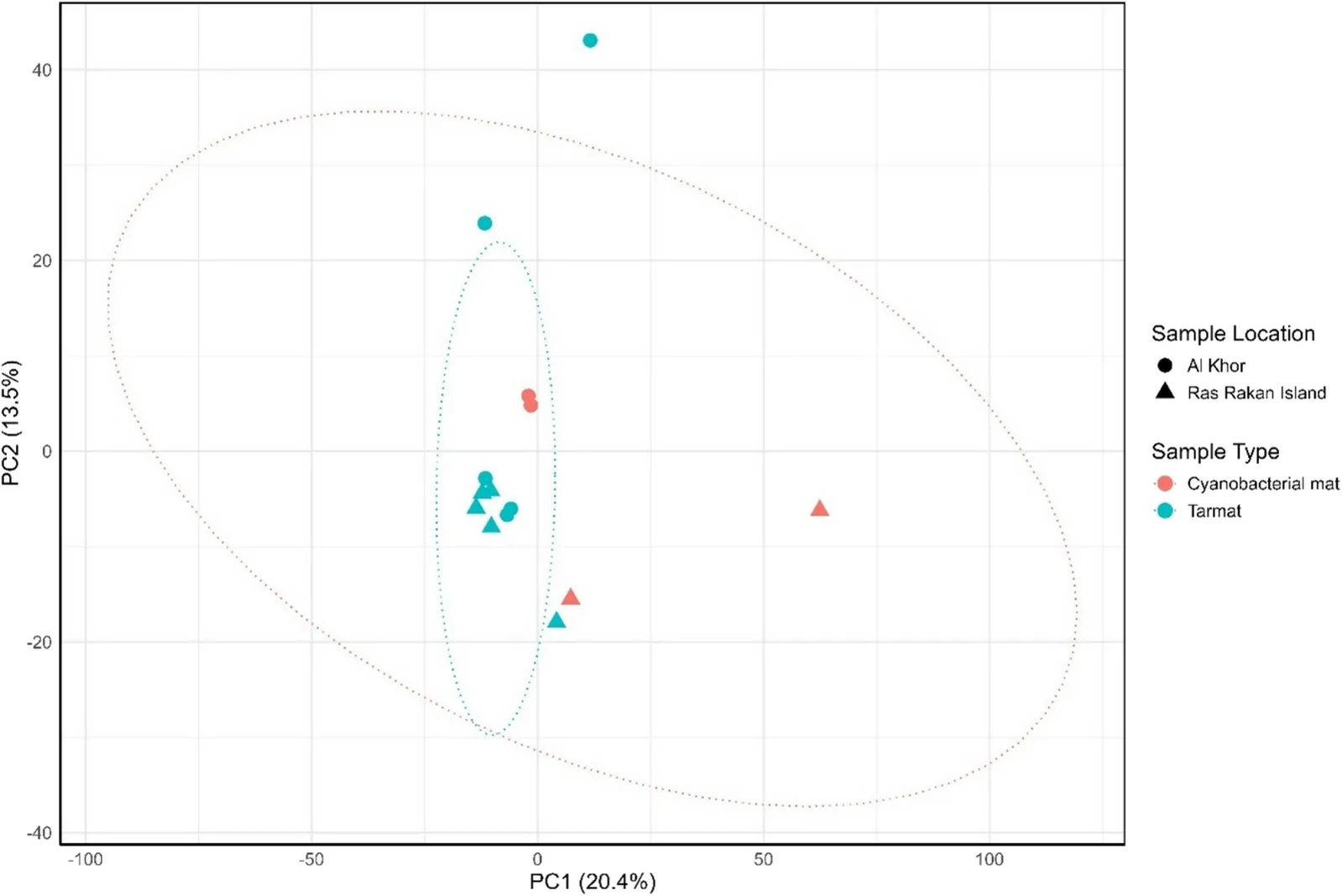
PCA plot showing the bacterial composition across sample type and location and the percentage of variance PC1(20.4%) and PC2 (13.5%)

The alpha diversity measurements showed that there was a variation in Shannon and Species richness, especially cyanobacterial mat samples have higher species richness and Shannon index than tar mat samples (Fig. 4). The species richness in Ras Rakan varied between 119 and 747 taxa while in Al Khor ranges from 200 to 526. Shannon index ranges from 2 to 5.4 in Ras Rakan compared to Al Khor (3.6 to 5.4). However, there was no significant difference in the species richness of tar mat and cyanobacterial mat samples (Kruskal-Wallis chi-squared = 3.38, df = 1, p-value = 0.06599). Similarly, Shannon index showed no significant differences of both sample types (Kruskal-Wallis chi-squared = 2.88, df = 1, p-value = 0.08969) collected from Ras Rakan and Al Khor (Wilcoxon rank sum test *p* > 0.1) (Fig. 4). Principal Component Analysis (PCA) was performed on bacterial community composition between the sampling locations (Ras Rakan and Al Khor) and sample types (tar mat and cyanobacterial mat). The PCA result revealed that PC1 showed the variation of 20.4% and PC2 showed the variation of 13.5%. PERMANOVA based on Euclidean distances showed that tar mats and cyanobacterial mats significantly influenced the bacterial community composition (R2 = 0.190, F = 3.7, *p* = 0.036). (Fig. 5).

**Fig. 6:**
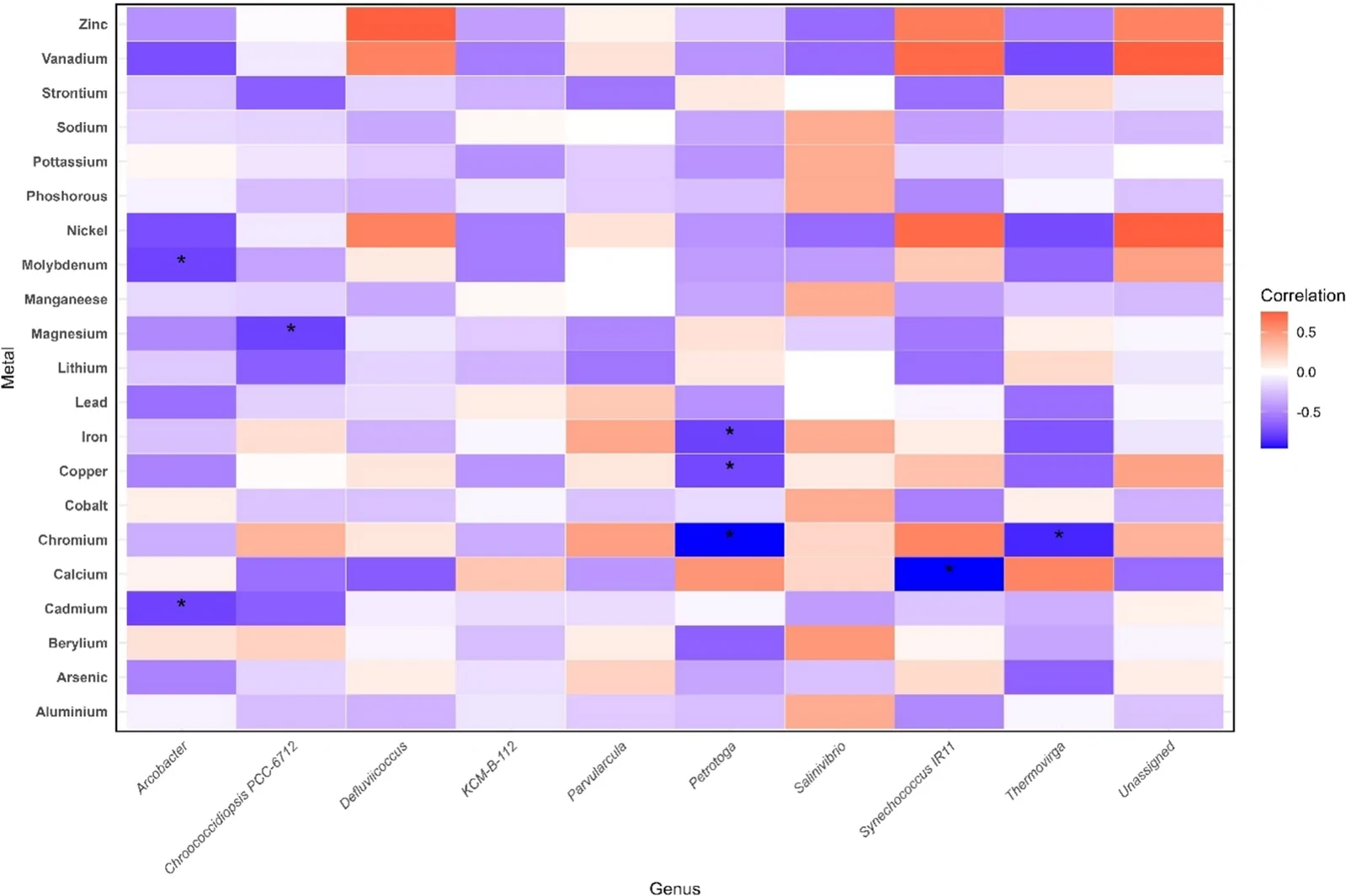
Heat map showing the Spearman correlation matrix based on the top 10 abundant genera and metal concentrations at Ras Rakan Island, (*) mark statistically significant correlations (*p* < 0.05)

Spearman correlation matrices were calculated to reveal the relationship between heavy metal concentrations and the top 10 abundant genera in Ras Rakan Island (Fig. 6). The Spearman correlation analysis revealed that although genera from Ras Rakan showed positive correlations with various metals, we did not find any significant correlations. In contrast, we found several genera that show significant negative correlations with specific metals (*p* < 0.05). For example, *Arcobacter* showed a negative correlation with Cd, *Chroococcidiopsis* PCC-6712 showed negative correlation with Mg, and the genus *Petrotoga* showed negative correlation with Cr, Cu and Fe. Other genera such as *Thermovirga* also showed significant negative correlation with Cr (*p* < 0.05).

## Discussion

### Elemental Distribution in Tar Mat and Cyanobacterial Mat Samples along the Ras Rakan Island

In the present study, the elemental distribution from tar mat samples from the Ras Rakan shows varying concentrations, particularly in association with specific sample types. The wide range of tar samples analyzed, such as cyanobacterial mats, soft and hard tar mats, and tar mats embedded with plastics, reflect the complex characteristics of the local environment. The cyanobacterial mats with hydrocarbon layers, interacting with seawater might considerably influence the metal concentrations across tar mat types [18]. The highest concentration was recorded for Ca (227205.89 µg g-1) in Sample RR-T-3, a soft tar mat deposited on the rock. However, samples RR-C-1, followed by RR-T-1 and RR-T- 4, also showed higher concentrations for several metals, particularly Al, Ca, Fe, and Na (Table 2). Al, Ca, Fe, and Na are dominant crustal elements, and their enrichment in the studied tar mats is consistent with the sedimentary geological framework of Qatar. The prevalence of these elements is further enhanced by intense atmospheric dust deposition, a major regional process that contributes substantial Ca and associated crustal elements to coastal sediments [19]. Additionally, seawater intrusion promotes Na accumulation in sediments, which can be gradually transferred to overlying tar mats through prolonged sediment–oil interaction. Fe in tar balls may occur as suspended inorganic compounds or organometallic complexes [20]. Elevated Fe levels in tar balls can also be linked to corrosion processes in oil storage tanks or extraction components [21], which release iron into the environment, increasing localized concentrations. Under anoxic conditions, Fe³⁺ oxides undergo reduction, releasing dissolved Fe²⁺ and thereby increasing Fe concentrations in the water. The observed increase in Fe concentration coupled with a decrease in Mn concentration also indicates redox-driven geochemical partitioning, where the reduction of Fe³⁺ is thermodynamically more favorable and occurs before the reduction of Mn⁴⁺ [22]. The cyanobacterial mats RR-C-1, RR-C-2, and RR-CT-5 showed notable differences in elemental concentrations, which can be attributed to the specific sample type and site-specific environmental factors such as direct seawater exposure, hydrocarbon layer presence, and sediment composition. The cyanobacterial mat RR-C-1 collected directly from seawater contained relatively higher concentrations of most of the elements, including Al, Fe, Mg, Na, Mn, Cr, V, Pb, Cd, and Zn, than RR-C-2, which were developed on the hydrocarbon layer. This could be due to the enhanced binding capacity of cyanobacterial mats by active biosorption through extracellular polymeric substances and prolonged interaction with element-bearing seawater and suspended particulates [23]. Cyanobacteria can synthesize metal-binding proteins called metallothioneins, often associated with Zn, Cu, Cd, and Hg [23]. In contrast, the hydrocarbon-layered mat RR-C-2 showed lower crustal element concentrations but increased petroleum-related metals Ni and V and lower concentration of Cd and Pb, indicating hydrocarbon presence alters elemental composition by limiting sediment contact and modifying redox and sorption conditions within the mat. The underlying hydrocarbon layer RR-CT-5 contained minimal concentrations of major elements like Al, Fe, Mn, Na, and P, reflecting low metal retention in the oil-dominated matrix, while higher relative levels of Ni, V, and Zn suggest these metals preferentially associate with petroleum compounds and resist weathering. Cu, Fe and Zn are essential micronutrients required in small quantities for cyanobacterial metabolic processes and enzyme function. However, elevated concentrations may exert inhibitory effects. In contrast, metals such as Cd, Pb, Cr, and As are toxic even at low concentrations [24]. The increased presence of Pb in the cyanobacterial mat samples may pose potential toxicity risks to associated cyanobacterial communities, as Pb can persist through transformation into lead sulfate and interactions with organic matter [25].

Elemental concentrations in the tar mat samples were compared with values reported for tar residues from other regions (Table S2), revealing generally higher concentrations for several elements relative to previous studies. In contrast, lower elemental concentrations have been reported for tar balls along the Nigerian coastline [26] and the southern Caspian Sea [20], while tar balls from Alexandria, Egypt showed increased V and Mg concentrations [27]. Petroleum related elements Ni and V are relatively resistant to weathering and are widely used as geochemical indicators to petroleum-derived pollution [28]. Although source identification is not the primary focus of this study, V/Ni ratios were examined to provide contextual insight. Previous studies have reported that specific Saudi Arabian crude oil and Iranian Nowruz crude oil had V/Ni ratios of 4.35 and 4.40, respectively [29, 30]. An earlier study by Literathy and Foda [31] reported a V/Ni ratio of 2.9–3.2 for Kuwait crude oil slicks, which is comparable to the V/Ni ratios observed in the current tar mat samples (Table S3) These ratios are higher than those reported for tar balls from the southeast Caspian Sea [20]. Oils with V/Ni ratios ≥ 1.0 are generally indicative of a marine organic origin [32]. The relatively stable V/Ni ratios observed in this study suggest persistence of these metal signatures despite prolonged weathering.

### Bacterial Diversity and Elemental Association

The bacterial community structure of the tar mat and cyanobacterial mat significantly differed between the two sampling locations. The cyanobacterial composition also suggested a strong correlation with elemental concentrations. Several studies have identified Proteobacteria as the key players in hydrocarbon degradation [33–35]. Recently, Dell’Anno et al. [36] reported distinct cyanobacterial assemblages inhabiting marine sediments polluted with polycyclic aromatic hydrocarbons and heavy metals. The relative abundance of the class Gammaproteobacteria is substantially present in all samples of Al-Khor and Ras Rakan tar mat and cyanobacterial mat (Fig. 2). Earlier studies have reported Gammaproteobacteria as a potential bioindicator group associated with the degradation of polycyclic aromatic hydrocarbons [37].

In the present study, higher relative abundance of class Bacteroidia in cyanobacterial mats (Fig. 2) is possibly associated with the increase in heavy metals concentration. A previous study documented the abundance of phylum Bacteroidetes in tar ball samples [37]. In addition, the dominant KCM-B-112 clade observed in the tar mat samples aligns with its previously reported dominance in petroleum-contaminated soils [38]. Our findings demonstrate a positive correlation of elements with various taxa, including *Synechoccocus* sp. However, further experiments are needed to determine the interaction of such heavy metals with diverse bacterial communities. It is well documented that cyanobacteria harbor genes for the biosorption of heavy metals. Studies have shown that cyanobacteria are highly efficient in degrading crude oil components when they form consortia with related heterotrophic bacteria within the cyanobacterial mats [39, 40]. These cyanobacterial communities are considered as the major environmental indicators and they respond more rapidly to environmental deterioration. Therefore, it is highly crucial to understand the association between the cyanobacterial diversity of both cyanobacterial mats and tar mats.

Several bacterial species, such as *Alcanivorax* and *Marinobacter* were also found in low relative abundance in the tar mat samples suggesting their potential ability to metabolize the heavy metals and hydrocarbons present in oil contaminant sites [41]. Nevertheless, direct evidence is needed to link the changes in bacterial community dynamics and high concentrations of heavy metals. The relative abundance of *Petrotoga* was found in high abundance in one tar mat samples from Al Khor compared to Ras Rakan samples. This genus was previously isolated from petroleum reservoirs, indicating a potential role in fermenting complex organic compounds [42]. A high abundance of *Salinivibrio* sp. was recorded in Ras Rakan Island, supporting their previously reported increased tolerance to heavy metals [43, 44]. Several studies have shown that heavy metals notably influence the bacterial dynamics in marine polluted sites [45–48].

## Conclusions

The Gulf faces a constant threat of marine oil pollution, with the formation of tar mats representing the ultimate fate of oil residues from natural and anthropogenic resources. The analysis of metal concentrations in Ras Rakan Island tar mat samples exhibited a high concentration of essential elements such as Ca, Mg, Al, Fe and K and low concentration of heavy metals including As, Be, Cd, Pb and Zn, indicating an overall minimal anthropogenic influence in the region. The bacterial communities at Ras Rakan showed differential relative abundances of unique genera compared to those at Al Khor. The distinct bacterial communities at Ras Rakan correlated positively with heavy metal presence, suggesting their crucial ecological role in metal-rich environments. Nevertheless, the current study also indicates that Ras Rakan Island is an essential reference point for determining initial metal levels in coastal ecosystems unaffected by anthropogenic activities. In addition, V/Ni ratios were consistent across weathered tar mats, suggesting a common marine source.

The focus on cyanobacterial communities associated with tar residues, particularly their hydrocarbon-degrading potential, reflects a growing interest in bioremediation strategies for oil-polluted ecosystems. In this study, we investigated the bacterial diversity associated with tar mats and cyanobacterial mats, along with their elemental composition. Although we observed distinct differences in community composition between the two sites, it is crucial to acknowledge that 16 S rRNA amplicon sequencing captures only the taxonomic structure of these communities and does not directly assess their functional activity or metabolic potential. Furthermore, the associations between bacterial taxa and elemental concentrations could only be evaluated for Ras Rakan samples, which constrains our ability to fully comprehend how cyanobacterial communities interact with various elements. Expanding the analysis to include additional sites, sediment samples, and further measurements such as PAH quantification would provide a more comprehensive understanding of cyanobacterial-environment interactions. Despite these limitations, this study offers valuable baseline information on bacterial communities associated with tar residues and cyanobacterial mats in the Gulf, highlighting patterns that remain largely unexplored and establishing a foundation for future research into the ecological roles and bioremediation potential of these communities. Future studies need to incorporate the functional metabolism of bacteria and their interactions with environmental conditions associated with marine pollution.

## Supplementary Information

Below is the link to the electronic supplementary material.

Supplementary Material 1 (DOCX 5.32 MB)

## Data Availability

The raw nucleotide sequence dataset was deposited in the NCBI Sequence Read Archive (SRA) under accession PRJNA714822.
