# Supplementary material for: Taxonomic Profiling of Bacterial Communities Associated with Metals in the Tar Mats and Cyanobacterial Mats of the Qatar Coast, Arabian Gulf
Source: Microb Ecol. 2026 Apr 24;89(1):123. doi: 10.1007/s00248-026-02761-y (PMC13246544; doi:10.1007/s00248-026-02761-y)
Supplement: Supplementary file 1 — Supplementary Material 1 (DOCX 5.32 MB) [file 248_2026_2761_MOESM1_ESM.docx]

**Table S1**

Overall average physico-chemical parameters of water from sampling locations

| Al Khor | Site | Sampling coordinates | Temp (^◦^C) | DO (mg/L) | Salinity (ppt) | pH | NTU | Chl-*a* (µg/L) |
| --- | --- | --- | --- | --- | --- | --- | --- | --- |
|  | site 1 | 25º40'04.3"N 51º32'02.9"E | 32.62 | 6.70 | 42.85 | 8.50 | 14.76 | 0.59 |
|  | site 2 | 25º40'03.2"N 51º32'11.9"E | 32.72 | 6.68 | 42.74 | 8.54 | 50.61 | 1.04 |
| Ras Rakan | site 1 | 26º10'57"N 51º12'49"E | 33.66 | 6.56 | 43.46 | 8.00 | 0.36 | 0.24 |
|  | site 2 | 26º10'50"N 51º14'36"E | 33.66 | 6.21 | 43.41 | 7.95 | -0.05 | 0.22 |
|  | site 3 | 26º10'48"N 51º14'38"E | 33.68 | 6.68 | 43.57 | 8.00 | 0.09 | 0.06 |

**Table S2**

Concentrations of metals (µg g^-1^) in tar residues compared with previous studies

| Reference | Fe | Mn | Cu | Ni | Co | Mg | Cr | Pb | V | V/Ni |
| --- | --- | --- | --- | --- | --- | --- | --- | --- | --- | --- |
| Current study | 247.61-1267.58 | 0.46-273.69 | 1.28-181.6 | 5.31-196.99 | 0.35-180.15 | 451.95-17934.83 | 0.22- 203.67 | 0.3-183.8 | 5.08-200.93 | 0.96-3.19 |
| [26] | 0.3-1.99 | 0.06-0.28 | 0.13-0.39 | 0.15-0.66 | ND | ND | ND | 0.63-1.70 | 1.69-69.44 | 0.95-1.00 |
| [49] | ND | ND | ND | 8.6- 59.8 | ND | ND | ND | ND | 25.4-165.0 | 2.9 ± 0.01 |
| [27] | 13.36- 40.45 | ND | 0.19- 9.30 | 11.17- 34.25 | 0.06- 5.03 | 37.00- 198.51 | ND | ND | 77.65- 168.25 | 2.66- 10.94 |
| [50] | ND | ND | ND | 0.24 -8.46 | ND | ND | 0.49 -4.75 | ND | ND | ND |
| [20] | 56.06-78.35 | 5.88-62.33 | 1.19-  9.98 | 0.67- 2.57 | 0.38- 1.93 | ND | ND | ND | 0.72- 3.48 | 1.07-1.47 |

ND – No data

**TABLE S3**

V/Ni ratios of tar mat and cyanobacterial mat samples of Ras Rakan Island

| **Analytes** | **V (µg g^-1^)** | **Ni (µg g^-1^)** | **V/Ni**  **(Current study)** |
| --- | --- | --- | --- |
| RR-T-1 | 5.08 | 5.31 | 0.96 |
| RR-C-1 | 200.93 | 196.99 | 1.02 |
| RR-T-2 | 134.46 | 45.71 | 2.94 |
| RR-T-3 | 35.1 | 11 | 3.19 |
| RR-T-4 | 36.92 | 13.89 | 2.66 |
| RR-C-2 | 169.95 | 81.64 | 2.08 |
| RR-CT-5 | 82.52 | 27.82 | 2.97 |

**Fig. S1** Tar mat and cyanobacterial mat samples collected from Ras Rakan


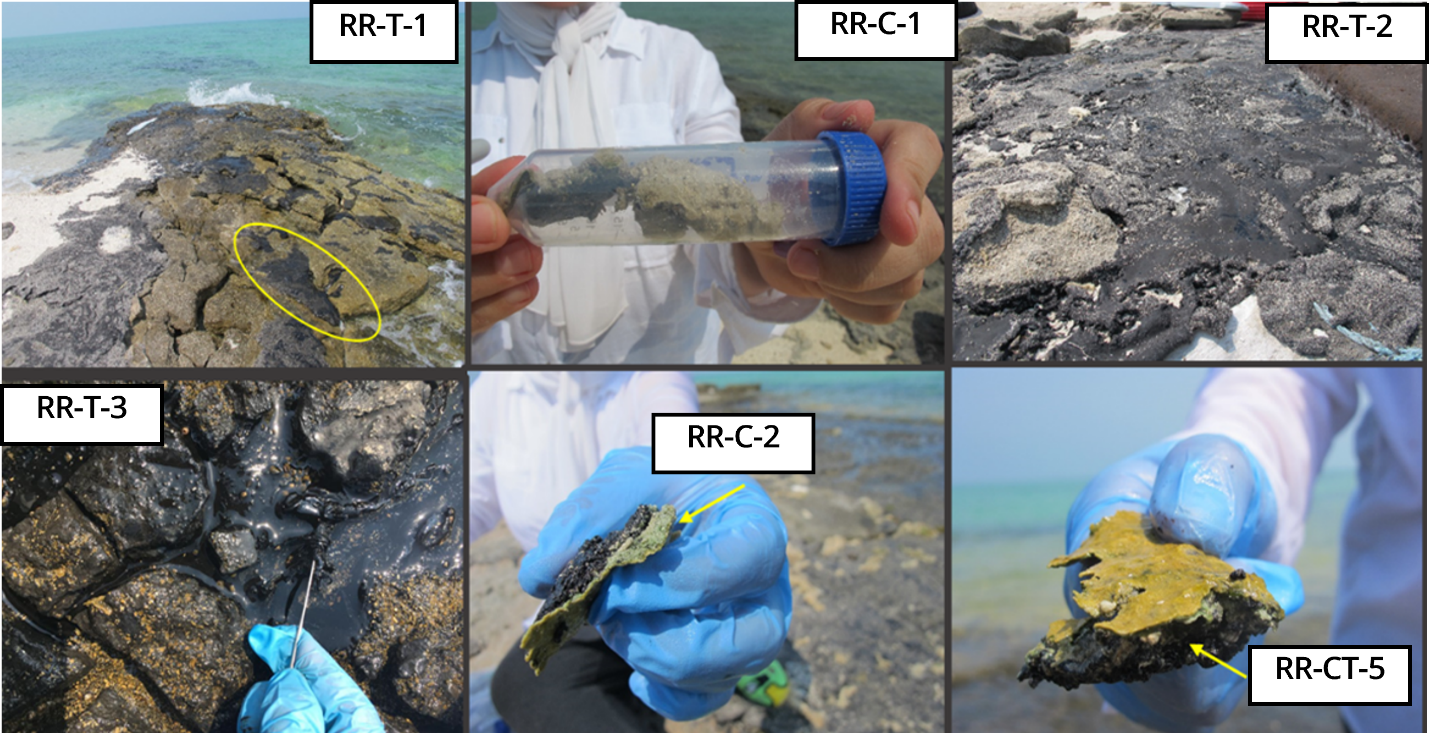


**Fig. S2** Tar mat and cyanobacterial mat samples collected from Al Khor


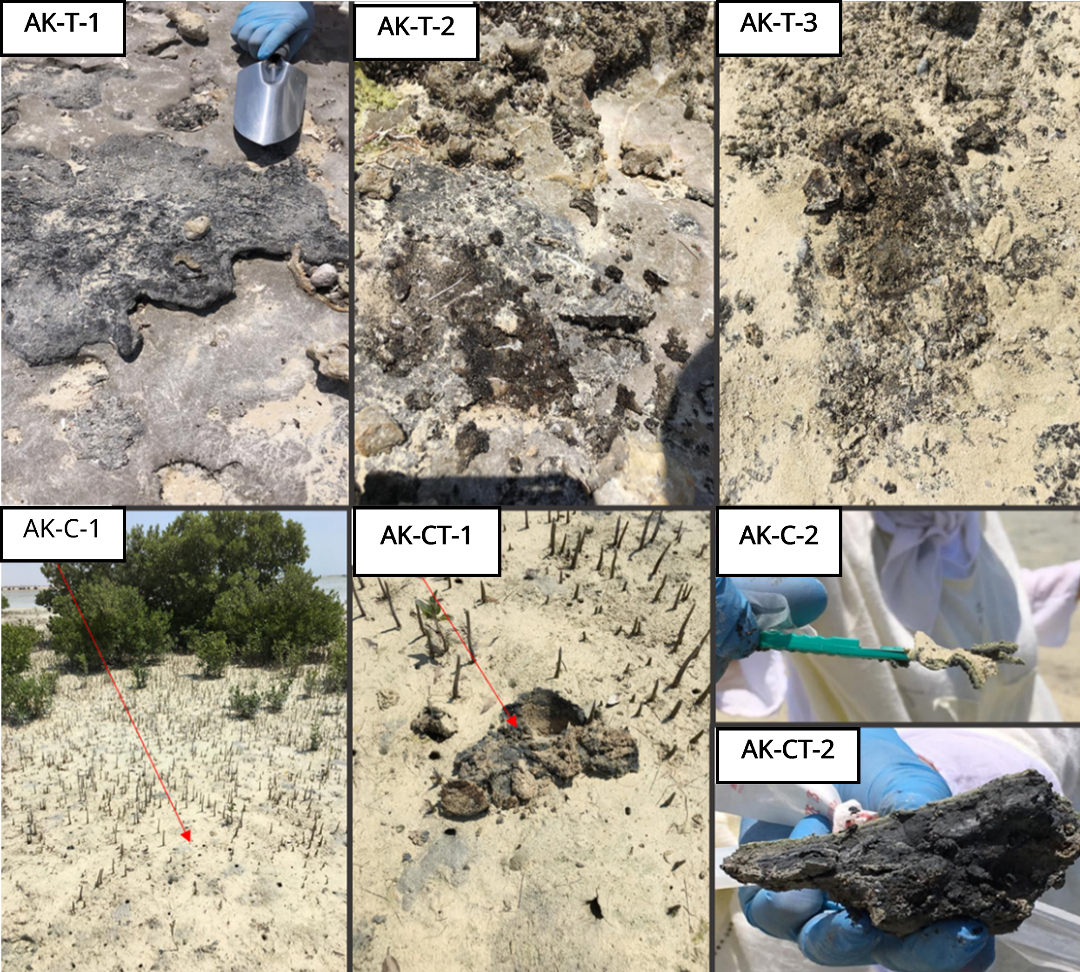


**Fig. S3**

Boxplot showing the overall relative abundance of bacterial genera at Al Khor and Ras Rakan


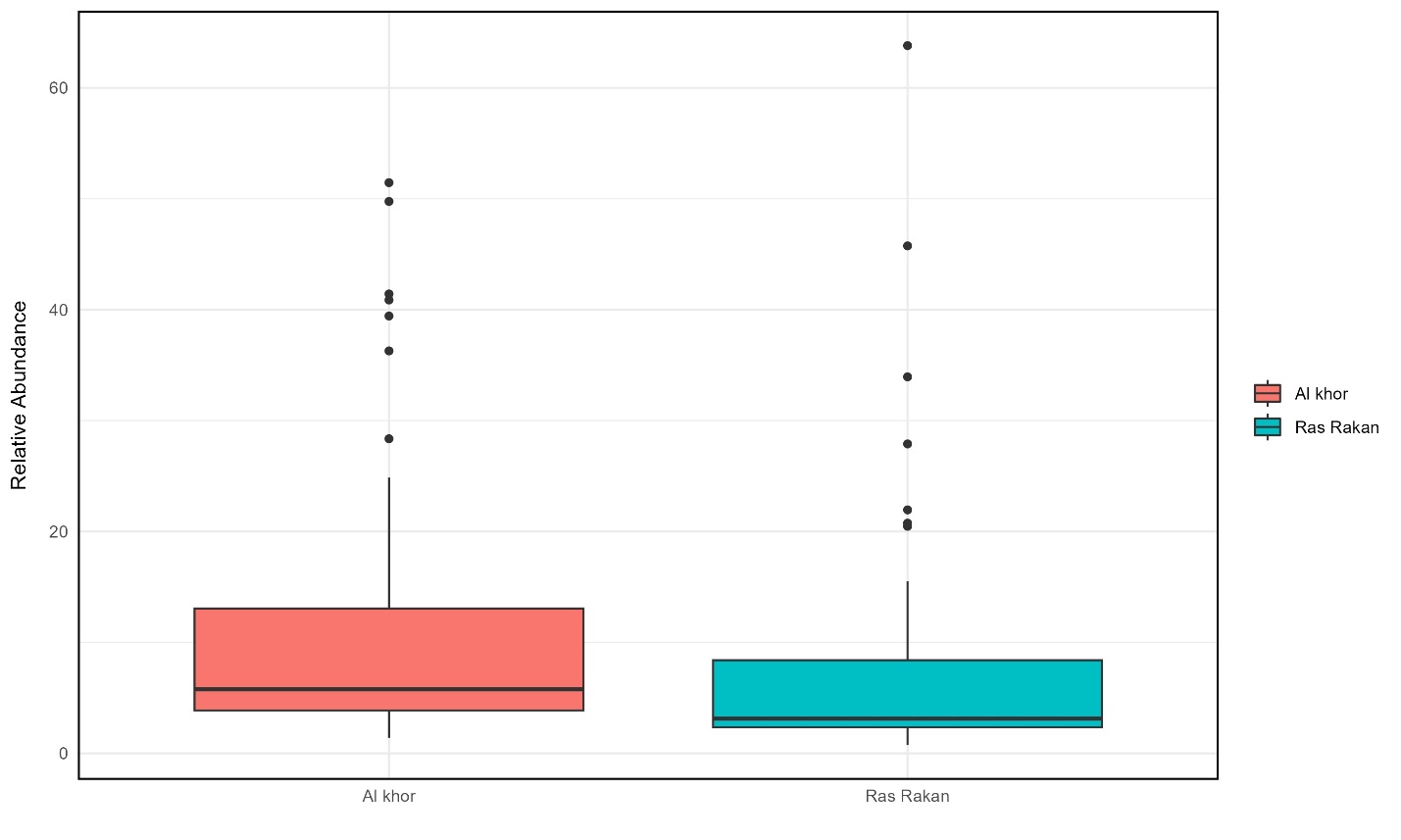


**References**

[49] Lavilla I, Vilas P, Millos J, Bendicho C 2006 Development of an ultrasound-assisted extraction method for biomonitoring of vanadium and nickel in the coastal environment under the influence of the Prestige fuel spill (North east Atlantic Ocean). Analytica Chimica Acta, 577(1), 119-125. <https://doi.org/10.1016/j.aca.2006.06.021>.

[50] Wise Jr JP, Wise JT, Wise CF, Wise SS, Gianios Jr C, Xie H, Wise Sr JP 2014 Concentrations of the genotoxic metals, chromium and nickel, in whales, tar balls, oil slicks, and released oil from the gulf of Mexico in the immediate aftermath of the deepwater horizon oil crisis: is genotoxic metal exposure part of the deepwater horizon legacy? Environmental science & technology, 48(5), 2997-3006.
